# Modeling of electromagnetic propagation inside jet engine environment using statistical electromagnetic approach

**DOI:** 10.1038/s41598-024-53529-8

**Published:** 2024-02-13

**Authors:** A. Krishna, A. F. Abdelaziz, T. Khattab

**Affiliations:** 1 Department of Electrical Engineering, Qatar University, Doha, Qatar; 2 Department of Electrical and Communication Engineering, Faculty of Engineering, Cairo University, Giza, Egypt

**Keywords:** Engineering, Electrical and electronic engineering

## Abstract

In this paper, the distribution of the electromagnetic field inside a complex jet engine environment is analyzed using statistical electromagnetics. The jet engine environment is an extremely complex geometry and exhibits random behavior due to the presence of moving metallic parts. This renders traditional analytical and simulation modeling techniques highly inefficient. To address this issue, two different approaches are proposed to model the propagation characteristics of the jet engine environment. The first is an innovative dynamic system approach based on dynamic system simulation which is inspired by the analysis of mechanically stirred reverberation chambers. In the dynamic system simulation the dynamic system, which is characterized by the rotation of a distinct set of blades, is primarily studied through the simulation program. A dimension scaling method is also introduced along with the dynamic system simulation to solve the complex jet engine environment. In the second approach, a novel statistical excitation method is applied to develop an equivalent model for the dynamic jet engine system. The studied jet engine is considered as a static jet engine system without blade rotation (static blades), but with a random excitation.A small signal analysis method is used to integrate the static and dynamic system parameters to generate random excitation characteristics of the static system. The extracted electric field values from the dynamic jet engine simulation environment and the static system field values from the small signal analysis have been analyzed statistically to prove the statistical equality between the two systems. The numerical results of the static system model are presented and verified through comparison with finite element method simulation packages.

## Introduction

**Figure 1:**
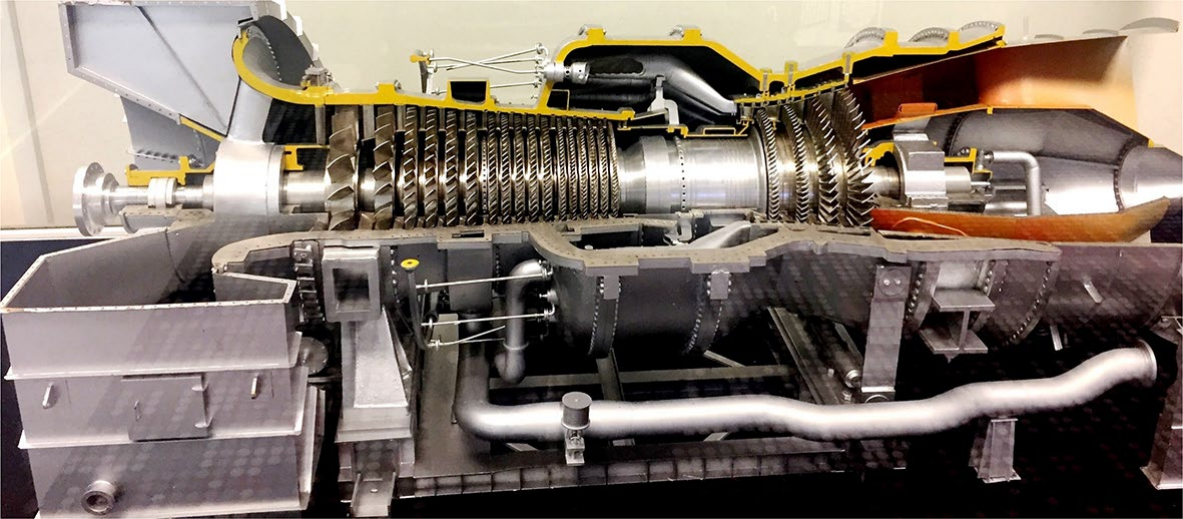
Photograph of jet engine model (@ Qatar University).

The modeling of the electromagnetic (EM) propagation inside the complex jet engine environment is gaining momentum, due to the increasing interest in measuring parameters that earlier were impossible or difficult to measure with wired telemetry systems. The jet engine environment is considered as an unreliable and unbounded cavity propagation environments due to their complex geometry and the presence of moving metallic parts with in the propagation path as shown in Fig. 1. The metallic jet engine environment, the dynamic rotation of the fan blades and the stators will affect the performance of the EM propagation. Thus, analysis of electromagnetic propagation inside the turbine is very important for the installation of wireless sensor in applications such as radar cross-section (RCS) reduction, and communication channel modeling. Moreover, to monitor the environment attributes such as temperature, pressure, strain, etc.; and to ensure the personal and environmental safety, deployment of wireless sensor network (WSN) inside harsh environment is becoming more appealing due to high cost of material and maintenance of wiring^1–3^. A WSN can been positioned inside the harsh environment and then it can transmit the information to a receiver (Rx) placed outside the environment wirelessly. Recent trend in aviation industry considers the utilization of wireless sensing inside jet engines for the same purposes of reduction of wiring cost and maintenance time. This trend is called fly-by-wirelss^1,2^. In many cases, the behavior of the wireless communication channel over which this information is transferred is not well understood, especially for harsh environments like jet engines, where the typical channel models are not applicable due to dynamics resulting from rotating metallic parts. Studies have considered the mechanical design of blades to increase efficiency and resilience in harsh environment^4,5^. These designs require continuous sensing for structural health monitoring, which can utilize wireless sensing networks. Hence, it is highly important to study the characteristics of wave propagation through the complex and harsh environment to develop a provably reliable wireless communication system inside such environment. The concepts presented here are not confined to jet engines and gas turbines alone, as these issues can be generalized for the study of electromagnetic propagation inside any large, complex-geometry cavities containing rotating metallic objects. Only very few research have been done on the EM propagation within jet engines, particularly in terms of sensor applications. The lack of comprehensive studies in this area suggests that a substantial amount of analysis is required to achieve a full understanding of the jet engine environment so that WSN can be installed in a jet engine. Researchers are facing many challenges to determine the field distribution inside such complex jet engine environment by using deterministic approach. Thus, we proposed statistical electromagnetics (STEM) as an alternative study to existing numerical approaches to characterize the jet engine environment. The concept of STEM and reverberation chamber approach are applied for many configurations where large cavity with complex environments are encountered^6–10^. STEM represents an innovative methodology that adeptly captures and models EM behavior within intricate and dynamic environments, without dealing with the complex details. This methodology addresses the problem of treating interior responses of complex field enclosures or systems by modeling the problem using a probabilistic approach. Reverberation chamber (RC) is the classical application of STEM^11,12^.

In order to take into consideration the challenges of the analysis of EM propagation inside harsh EM environments, a probabilistic approach based on STEM is proposed as a novel approach for analyzing the jet engine environment, without dealing with the complex details inside the engine cavity^13^. This method helps in expecting the field precisely inside the medium, which has very small and large dimensions simultaneously. However, when analyzing the jet engine model with rotating blades by using STEM approach, we must consider the effect of different factors, such as number of blades, number of rotations, orientation of blades, frequency of operation, Doppler effect etc.^14,15^. Moreover, analyzing the whole jet engine blade rotation is time consuming and needs a more complex simulation procedure^13,14^. Hence, Statistical Excitation (SE) concept was proposed as an efficient approach for analyzing jet engine environment by Krishna et al.^16^. The concept of SE has been adopted from statistical antenna (SA) hypothesis which has been applied in many applications to characterize the natural randomness that arises during the design or manufacturing process of large antenna systems^17–20^. However, we adopt SA concept and propose a novel method to model the source of randomness of the medium instead of blade rotation inside jet engine environment.

### Jet engine EM analysis: state of Art

In this section, we have discussed latest state of art on different techniques found in the literature for the analysis of the electromagnetic wave propagation in jet engines. For last few years, researchers have been analyzing the scattering mechanism of electromagnetic waves in jet engine inlets especially open cavities or cavities embedded in a perfectly conducting infinite plane^21–27^. The main analysis methods found in the literature can be summarized as: analytical methods, numerical methods (low frequency, high frequency and hybrid method), experimental methods, and the STEM method^21,28, 29^. It is important to mention that all these proposed methods, except experimental and STEM methods, are mainly concentrated on the scattering analysis for the radar cross section reduction analysis^27,30–35^.

The analytical method has its constraints when it comes to representing the diverse characteristics of complex geometries. It often relies on simplified geometries, which can swiftly become intricate and less applicable to real-world structures. Experimental data of realistic jet engine structures is desired to study the physical EM interpretation of jet engines and the properties of internally propagating waves. Moreover, it is important to validate the reliability of numerical results through comparison against measured values. Early measurement based methods used scaled down jet engine models to obtain experimental data. The first test to analyze the propagation mechanism in jet engine used an experimental method to recognize field behavior for modeling the wireless communication channel inside the engine cavity^36^. To extract the field characteristics, several boreholes antennas are inserted among the various turbine stages and measurements are performed from inlet to the outlet and also from the outlet to those borehole antennas^36^. Subsequently, a half-scale jet engine model was developed by researchers for the experimental measurements to validate the feasibility of a radio communication channel inside the jet engine^37,38^. Although, all of them^36–38^ are working on a scaled down jet engine models to obtain experimental data and are dealing with different objective and system configurations compared to our objective. To the contrary of our considered system, the aforementioned experiments have part of the communication system mounted on the rotating blades. To the best of our knowledge, the experimental data available for a real jet engine are rare and challenging to access for safety and intellectual property restriction reasons^28^.

When considering the different analytical, numerical, and measurement methods used to analyze electromagnetic scattering from jet engine inlets and their components, it is important to note that, as the complexity of the jet engine increases, the existing deterministic techniques become less reliable and the need for the STEM approach becomes increasingly apparent^13,28^. However, it is essential to consider why STEM approach is suitable for a system like a jet engine turbine. This is primarily due to the inherent complexity of precisely evaluating the electric field characteristics in an electrically large system, which is dependent on various engine parameters. A deterministic approach for the complete analysis of such a complex system becomes exceptionally challenging due to its sensitivity to details such as cavity dimensions, material properties, and the frequency spectrum of excitation. The rationale behind the STEM approach lies in the argument that, to analyze the overall characteristics of the electric field within intricate cavities containing rotating metallic objects, a statistical analysis can be more effectively employed. In particular, such systems incorporate rich EM scattering with time variation, which renders the classical EM analysis prohibitively computationally expensive. The STEM approach successfully navigates the complexities of systems like the jet engine environment by adopting a probabilistic approach, thus avoiding the intricacies of the details inside the engine cavity^11–13, 28^. This method helps in expecting the field precisely inside the medium, which has a combinations of parts with small and large dimensions simultaneously.

A simplified jet engine model with a single stage of fan blade was developed and analyzed by using Ansys HFSS simulation tool. The analysis showed that the jet engine environment is similar to a mechanically stirred RC environment that develops a statistically uniform electrical field^13^. The squared magnitude of the electric field component follows an exponential distribution, when the antenna is at $\lambda$ distance from the center of the axis^13^. The primary analysis also proved that plane which is located at 66 mm away from the center axis can be considered as the best location for the receiving antenna as it is relatively far from the blades and completely satisfies the statistical theory. Later, the effect of the location of the excitation inside the engine was studied^14^. The analysis proved that jet engine environment has anisotropic and highly correlated field characteristics when the transmitter (Tx) antenna is located at a position other than $\lambda$^14^. A detailed study of different approaches applied for analyzing the jet engine environment is available in the literature^21,28^.

In those previous jet engine EM propagation analysis, the main objective was to extensively test the numerical validity of the hypothesis for a simple jet engine structure^13,14, 39^. In this journal, we present the EM propagation analysis from a segment model and theoretical back ground point of view.

- Two different models are proposed to characterize the EM propagation through the entire jet engine environment.
  1. Dynamic system model
  2. Static system model

In order to analyze the proposed system models, two different methodologies (approaches) are proposed. These approaches are proved as an efficient tool for analyzing complex jet engine environment into two different perceptive. Later, a novel mathematical analysis is proposed to model the statistical parameters of static system model in equivalence with the dynamic system model statistical parameters.

## Proposed jet engine EM analysis

The analysis of EM propagation through jet engine environment is considered as extremely challenging and complex due to two main reasons:

1. Complex large engine geometry
2. Harsh environment stemming from
  The existence of large number of scatterers
  Rotating blades

### Reduction of engine geometry complexity

**Figure 2:**
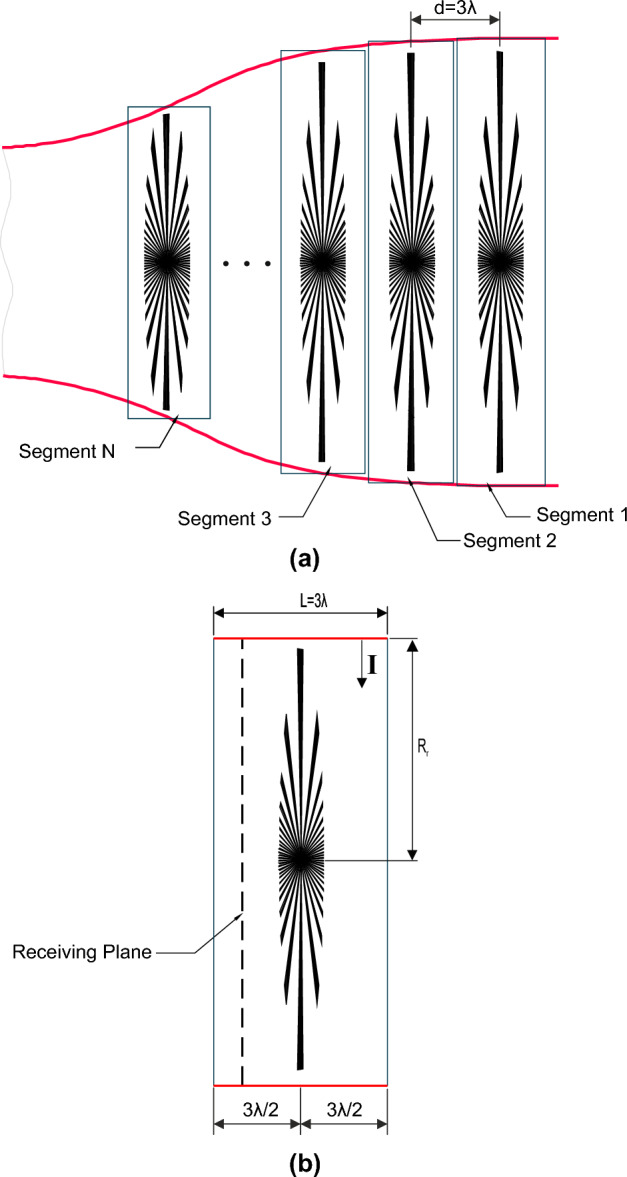
Schematic representation of Jet engine model: (**a**) segmentation of the complex jet engine model. (**b**) Details of single segment.

As explained before, the EM analysis through the complex jet engine environment is complex and tedious due to the huge engine dimensions. Thus, to generalize our analysis and to reduce the system complexity, a geometry reduction approach is proposed.

The jet engine environment is an extremely harsh environment as it contains curved surfaces and different stages of rotors and stators mounted on a central shaft as shown in Fig. 1. The rotational parts and the curved surfaces inside the engine environment make the environment more complicated for the analysis of propagation of EM field. Thus, to deal with the STEM approach and to analyze the entire jet engine environment, the engine has been decomposed into different segments, which is favorable to solve electrically large scale problems, as illustrated in Fig. 2a.

The jet engine segmentation is done, such that each segment is occupied by a single stage of a fan blade in the middle of the segment as shown in Fig. 2b. Later, the segments are cascaded in such a way that the output field of each segment will act as an input excitation for the next segment. However, in this analysis each jet engine segment is assumed to have an identical characteristics and hence, the characteristics of the EM propagation are analyzed through only one segment ($\textbf{segment 1}$). The most important requirement of this segmentation method is that the solutions of all the segments should be recombined (cascaded) to get a solution of the entire system. A more detailed analysis of the segments is available in the following sections.

### Targeting harsh environment

**Figure 3:**
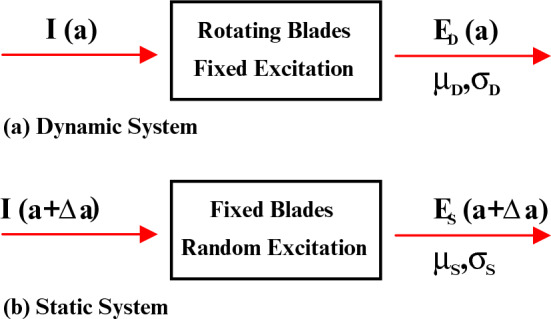
Block diagram of proposed system architecture: (**a**) dynamic system. (**b**) Static system.

In order to deal with the harsh jet engine environment with large number of scatterers, the $\textbf{segment 1}$ of the jet engine environment is modeled into two different perceptive (Approach1 and Approach2).

In Approach-1 (dynamic system approach), the jet engine environment is modeled as a dynamic system, where the dynamic rotation of the blades provide the necessary randomness inside the medium as shown in Fig. 3a. The excitation of the dynamic system is characterized by a fixed amplitude (*a*) and represented as $I_{a}$. In the dynamic system, $E_{D}(a)$ is the magnitude of the electric field resulting from this fixed antenna excitation, *a*. The mean and the standard deviation of the electric field in the dynamic system are represented by $\mu _{D}$  and  $\sigma _{D}$; respectively.

In dynamic system approach (DSA), a combination of dynamic system simulation (DSS) and the dimension scaling (DS) method has been adopted to solve the EM characteristics through the entire jet engine. This extends the work in^13,14, 16^ by allowing the STEM methodology to analyze the entire jet engine environment using mathematical interpretations.

In Approach-2 (Static system approach), the jet engine system is modeled as a static jet engine system, where the blades made stationary and the necessary randomness is provided by random excitation as shown in Fig. 3b. In the static system, $E_{S}(a+\Delta a)$ represents the magnitude of the electric field due to the randomized excitation. The mean and the variance of the electric field in the static system is represented as, $\mu _{S}$ and $\sigma _{S}$; respectively. The static system approach (SSA) adopts combination of a novel statistical excitation (SE) method and an analytical method (AM) to solve the EM propagation through the entire geometry.

## System model and methodology

### Single segment model

The proposed $\textbf{segment 1}$ as shown in Fig. 2b is represented by an open cylinder with a single stage of coplanar rotating blades. The blades are mounted on a central shaft and are extended to the wall of the cylinder. The outer cylinder length (**L**) is 3 $\lambda$ and has a varying radius ($R_{r}$). The central shaft and the attached blades are located at $(3/2)\lambda$ from the center of the axis. A single frequency continuous wave excitation is used to excite the jet engine system internally and the field characteristics are extracted from a particular receiving plane which is approximately at $\lambda$ distance from the center of the axis. A simplified model of this proposed segment is modeled using Ansys HFSS to characterize the EM analysis.

### *Approach-1*: jet engine statistical EM modeling using dynamic system approach

The dynamic system approach uses both DSS and DS method to model the statistical characteristics of EM propagation through the entire jet engine environment. The algorithm 1 represents the DSA procedure. The DSS of simplified jet engine model has been analyzed previously^13,14, 28, 39^. Therefore, in this journal only the dimension scaling approach is considered.

**Algorithm 1:**
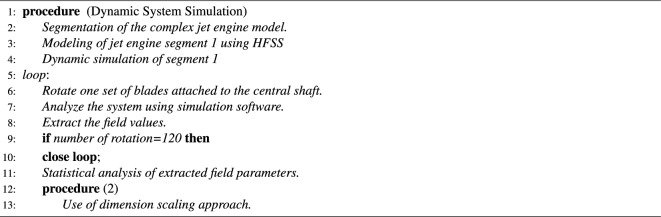
Dynamic system approach procedure

#### Dimension scaling method

DS method is an analytical approach that can be used to model the EM propagation through complex jet engine segments. The fundamental requirement to solve the propagation characteristics using DS method is that the jet engine geometry should be separable. As explained before, the jet engine environment is decomposed into different segments and each segment has a varying dimension.

Since, the jet engine environment resembles a mechanically stirred RC, all the statistical representations that have been evolved for the RC can be adopted to resolve the characteristics of the electromagnetic field inside the jet engine^13,28^.

The plane wave analysis of RC proved that, mean-square value of electric field component inside the complex cavity can be represented as in (1), where $E_{0}$ represents the constant amplitude of the field component^40^.

1 $$\begin{aligned} \left\langle {|E_{x}|}^2\right\rangle = \left\langle {|E_{y}|}^2\right\rangle = \left\langle {|E_{z}|}^2\right\rangle = \frac{|E_{0}|^2}{3} \end{aligned}$$

${|E_{0}|}^2$, can be represented in terms of cavity parameters as shown in (2), where $P_{t}$ is the transmitted power through the cavity, $\varepsilon$ is the free-space permittivity, ${\textbf{Q}}$ is the quality factor of the cavity, $\omega$ is the angular frequency and ${\textbf{V}}$ is the cavity volume^40^.

2 $$\begin{aligned} {|E_{0}|}^2={\textbf{Q}}P_{t} /\omega \varepsilon {\textbf{V}} \end{aligned}$$

From (1) and (2), the variance of the well stirred field component inside a complex cavity volume can be calculated as in (3).

3 $$\begin{aligned} {\sigma }^2 \approx {\textbf{Q}}P_{t} /\omega \varepsilon {\textbf{V}} \end{aligned}$$

i.e.;

4 $$\begin{aligned} {\sigma }^2\propto 1 / {\textbf{V}} \end{aligned}$$

From (3) and (4) it is clear that, the variance of the average electric field component is inversely related to the volume of the cavity. For our jet engine model; ${\textbf{V}} =\pi \mathbf {R_{r}}^{2} {\textbf{L}}$ and ${\textbf{L}}$ is considered as a constant for all the segments as shown in Fig. 1. Hence, (3) can be represented as;

5 $$\begin{aligned} {\sigma }^2 \propto 1 / \mathbf {R_{r}^2} \end{aligned}$$

Equation (5) shows the relation between the radius of the jet engine and the statistical parameter of electric field inside the jet engine environment. As the radius of the jet engine decreases, the variance of the electric field increases. The proposed dimension and variance relation is proved with the help of simulation results. This analysis is important to generalize the mathematical modeling of jet engine environment using STEM approach.

Thus, the DSA approach which constitutes both dynamic simulation and dimension scaling approach is an effective STEM approach to solve any complex jet engine model. In DSS method, the randomness of the medium was established by simulating the blades in different blade positions with equal angular increases. Hence, the efficiency of DSS requires an increased simulation positions with very small angular step size, which means increasing the time and the complexity of the computation. Moreover, the dynamic simulation may cause different kind of imperfections due to the analysis of field component close to any kind of metallic components. Also, the degree of uncertainty associated with the jet engine statistical parameters should also affect the accuracy of dynamic system simulations. Hence, to reduce these system complexities a static system approach is proposed as an alternative to the complex DSA method.

### *Approach-2*: Jet engine statistical EM modeling using SSA

As explained before, the SSA method uses a combination of statical excitation (SE) approach and mathematical modeling of the ideal well stirred jet engine cavity to analyze the field characteristics inside the entire jet engine environment. Algorithm 2 shows the flow of SSA procedure.

**Algorithm 2:**
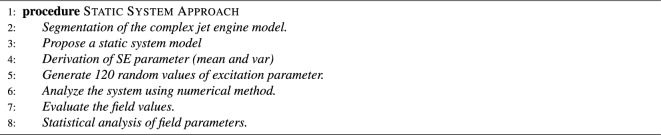
Approach 2 analysis procedure

#### Proposed statistical excitation model

SE method is introduced to develop an equivalent model for the fields generated from the dynamic system model. Thus, to deal with the SE method, we compare the field characteristics of two different jet engine system models. As mentioned before, in the dynamic system the blade rotation provides the necessary randomness inside the jet engine environment. In the dynamic system, $E_{D}(a)$ is the magnitude of the electric field resulting from the fixed antenna excitation, *a*. The mean and the standard deviation of the electric field in the dynamic system are represented by $\mu _{D}$  and  $\sigma _{D}$; respectively.

The static system represented in Fig. 3b has a random environment resulting from the random excitation (hence the use of SE) and stationary blades. The static system is excited by a constant deterministic antenna excitation, *a*, and another small random excitation, $\Delta a$ as given in (6). Hence, the random excitation of the static system is characterized by the random amplitude ($a+\Delta a$). As explained before, in the static system, $E_{S}(a+\Delta a)$ represents the magnitude of the electric field due to the randomized excitation. The mean and the variance of the electric field in the static system is represented as, $\mu _{S}$ and $\sigma _{S}$; respectively.

6 $$\begin{aligned} a_{\textrm{total}} = a + \Delta a. \end{aligned}$$

The primary aim of SE method is to have a statistical equality between the static and dynamic system. This is achieved by carefully choosing a random excitation variable ($\Delta a$), with a statistical distribution similar to that seen in the dynamic system model’s field distribution. In order to maintain the statistical equality between the static and dynamic system model and to translate the effect of dynamic blade rotation into random excitation, the statistical parameters of $\Delta a$; such as mean ($\mu _{\Delta }$), and variance ( $\sigma _{\Delta }^2$) have to be controlled. That means, the effect of rotation of the blades in the dynamic system should be translated into the random excitation in the static system. Consequently, instead of engaging in the computationally intensive simulation of blade rotation within the engine, the proposed SE approach intended to considering the jet engine as a static system. Hence, the engine is treated as static, without blade rotation but subject to a controlled random excitation. This new approach not only allows for a thorough exploration of system dynamics but also simplifies the computational challenges associated with simulating rotating blades.

## Mathematical analysis of SSA

In our earlier work^13,28^, we have established that the distribution of EM field intensity inside a jet engine follows a Gaussian distribution, which applies to both our proposed dynamic and static systems. What remain to be attained in this work is to ensure the parameters (mean and variance) of the Gaussian distribution governing the EM field intensity are the same in both systems. To maintain the statistical equality between the two systems, $\mu _{S}$ and $\sigma _{S}$ are assumed to be equal to $\mu _{D}$ and $\sigma _{D}$, respectively. The statistical parameters of $\Delta a$, i.e., $\mu _{\Delta }$ and $\sigma _{\Delta }$, are deduced accordingly, as shown in the following.

7 $$\begin{aligned} {{\textbf {E}}}[E_{D}(a)]= {{\textbf {E}}}[ E_{S}(a+\Delta a)], \end{aligned}$$

where ${{\textbf {E}}}[x]$ is the expected value of *x*.

8 $$\begin{aligned} {\textbf {Var}}[E_{D}(a)]= {\textbf {Var}}[ E_{S}(a+\Delta a)], \end{aligned}$$

where ${\textbf {Var}}[x]$ is the variance of *x*. Now, applying Taylor series expansion for $E_{S}(a+\Delta a)$ .

9 $$\begin{aligned} E_{S}(a+\Delta a) = E_{S}(a)+ \Delta a \frac{\partial E_{S}(a)}{\partial a} + \frac{1}{2}(\Delta a)^2\frac{\partial ^2 E_{S}(a)}{\partial a^2}+O(|\Delta a|^2), \end{aligned}$$

where $E_{S}(a)$ is the magnitude of the electric field for stationary blades due to the fixed excitation *a*. The value of $\Delta a$ is assumed to be very small compared to *a*; hence, we can use small signal analysis utilizing first order Taylor series expansion with first derivatives only to characterize the static system. Consequently,

10 $$\begin{aligned} E_{S}(a+\Delta a) \approx E_{S}(a)+\frac{\partial E_{S}(a)}{\partial a}\Delta a. \end{aligned}$$

Taking the expectation of (10), we get

11 $$\begin{aligned} {{\textbf {E}}}\left[ E_{S}(a+\Delta a)\right] = {{\textbf {E}}}\left[ E_{S}(a)\right] + E\left[ \frac{\partial E_{S}(a)}{\partial a}\Delta a\right] \end{aligned}$$

Since, *a* is a deterministic constant, ${{\textbf {E}}}[E_S(a)] = E_S(a)$. Accordingly, (11) can be rewritten as

12 $$\begin{aligned} {{\textbf {E}}}\left[ E_{S}(a+\Delta a)\right] = E_{S}(a) + \frac{\partial E_{S}(a)}{\partial a}{{\textbf {E}}}\left[ \Delta a\right] . \end{aligned}$$

From (7) and (12),

13 $$\begin{aligned} {{\textbf {E}}}[\Delta a] =\frac{{{\textbf {E}}}[E_{D}(a)] - E_{S}(a)}{(\partial E_{S}(a)/\partial a)} = \mu _{\Delta }. \end{aligned}$$

Later, from the definition of the variance of a random variable,

14 $$\begin{aligned} {\textbf {Var}}(X)={{\textbf {E}}}\left[ (X-{{\textbf {E}}}[X])^{2}\right] \end{aligned}$$

Applying (14) in (8),

$$\begin{aligned} {\textbf {Var}}[E_{S}(a+\Delta a)]= & {} {{\textbf {E}}}\left[ \left( E_{S}(a+\Delta a)-{{\textbf {E}}}[E_{S}(a+\Delta a)]\right) ^{2}\right] \\= & {} {{\textbf {E}}}\left[ \left( E_{S}(a)+\frac{\partial E_{S}(a)}{\partial a}\Delta a - E_{S}(a) - \right. \right. \\{} & {} \left. \left. \frac{\partial E_{S}(a)}{\partial a}{{\textbf {E}}}[\Delta a]\right) ^{2}\right] , \end{aligned}$$

i.e.,

15 $$\begin{aligned} {\textbf {Var}}[E_{S}(a+\Delta a)]= & {} {{\textbf {E}}}\left[ \left( \frac{\partial E_{S}(a)}{\partial a}\right) ^{2}\left( \Delta a-{{\textbf {E}}}[\Delta a]\right) ^{2}\right] \nonumber \\= & {} \left( \frac{\partial E_{S}(a)}{\partial a}\right) ^2{\textbf {Var}}(\Delta a). \end{aligned}$$

Substituting ${\textbf {Var}}[E_{S}(a+\Delta a)]$ from (8), we get

16 $$\begin{aligned} {\textbf {Var}}(E_{D}(a))= & {} \left( \frac{\partial E_{S}(a)}{\partial a}\right) ^2{\textbf {Var}}(\Delta a), \end{aligned}$$

17 $$\begin{aligned} {\textbf {Var}}(\Delta a)= & {} \frac{{\textbf {Var}}[E_{D}(a)]}{(\partial E_{S}(a)/\partial a)^{2}} = \sigma _{\Delta }^2. \end{aligned}$$

Equations (13) and (17), represents the statistical characteristics of $\Delta a$. From (13), and (17), it is clear that the mean value and the variance of the random excitation parameter depends on the mean and variance of the dynamic system electric field. Since, the jet engine environment is similar to a well stirred RC, the mean and the variance of the axial electric field components of the dynamic system can be analyzed using the **n** plane wave coupling method explained in^41^. In this paper, for the SE analysis we consider only a particular receiver location ($\theta =0^\circ$) and excitation location. Then, the mean value of the amplitudes of the field rectangular components and the mean square value of the field rectangular components resulting from the sum of **n** (**n** is a free parameter.) random plane waves can also be evaluated using this plane wave coupling approach as in (18) and (19)^41^.

18 $$\begin{aligned} \langle {|E_{D}|}\rangle= & {} E_{0}\cdot \sqrt{{{\textbf {n}}}}\cdot \sqrt{\frac{\pi }{12}} \end{aligned}$$

19 $$\begin{aligned} \langle {|E_{D}|^{2}}\rangle= & {} \frac{E_{0}^{2}\cdot {{{\textbf {n}}}}}{3} \end{aligned}$$

Later, using (13) and (17), 120 random samples of $\Delta a$ can be generated for a particular receiver location and this random samples are used to set up (6) to feed the static system model.

## Results

### *Approach-1*: dynamic system approach

**Figure 4:**
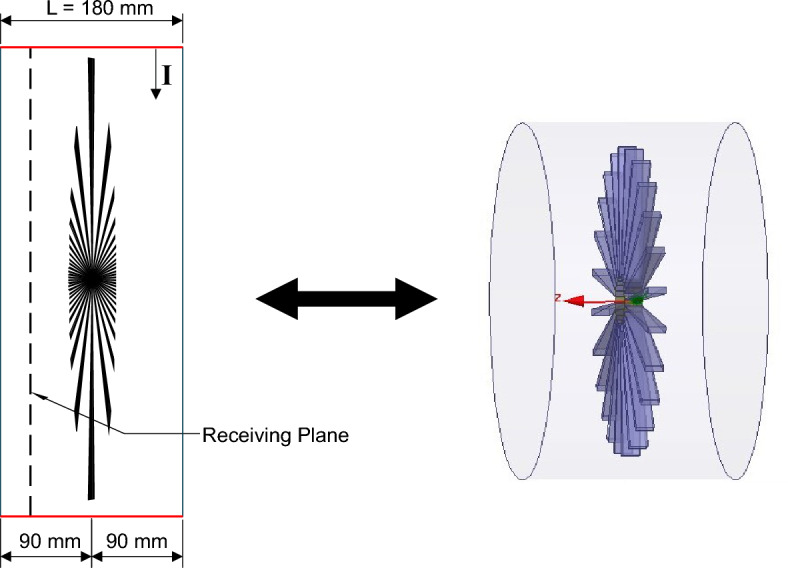
Simplified software model of segment1.

**Figure 5:**
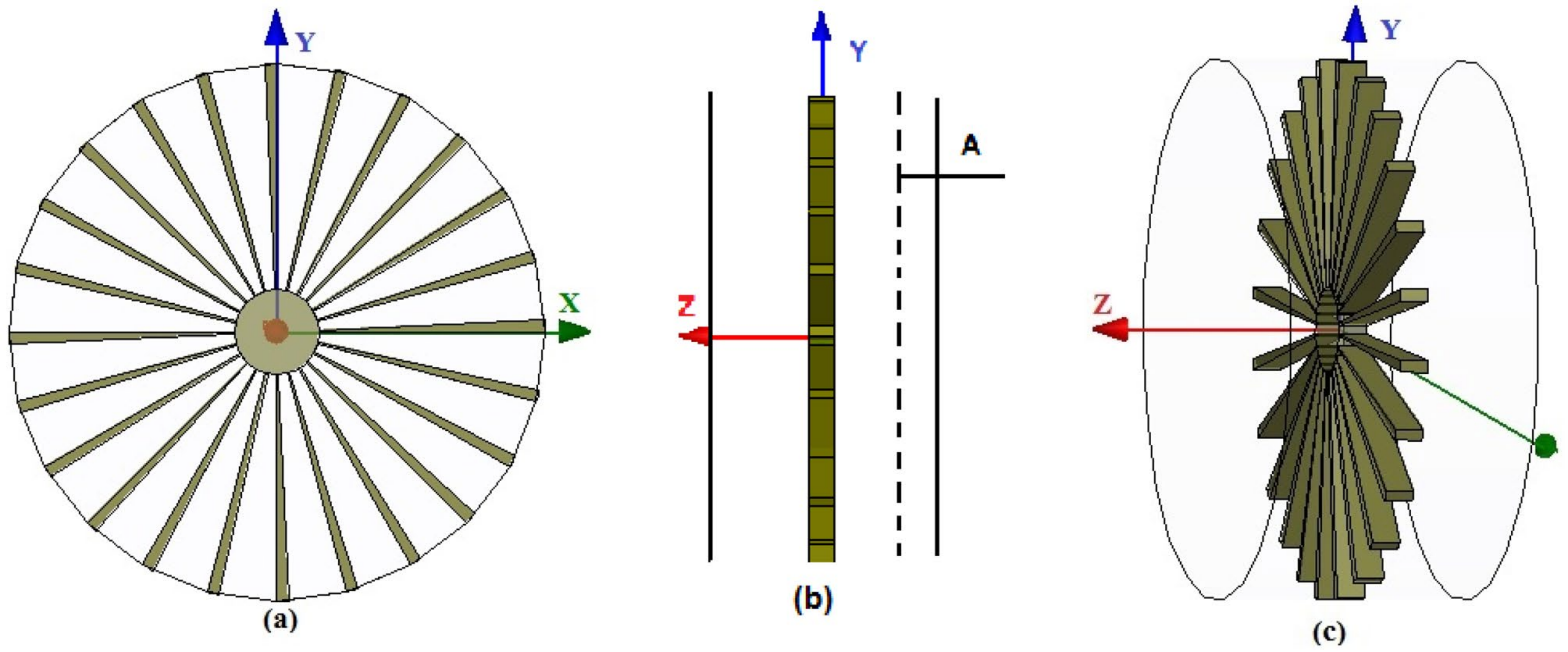
Simplified software model of segment1: (**a**) front view. (**b**) Side view. (**c**) 3-D engine view.

In order to prove the concept explained in DSA, a simplified scaled dynamic model of $\textbf{segment 1}$ is designed, and developed using HFSS full wave finite element method (FEM) simulation software as shown in Fig. 4^13,14^. The proposed model is represented by an open cylinder with a single stage of coplanar rotating blade consisting of 24 blades. The blades are mounted on a central shaft and the dimension of the central shaft is 60 mm in diameter and 20 mm in length. The angular thickness of the blade is 2.5$^\circ$. The outer cylinder length is 180 mm and its radius is 190 mm. The central shaft and the attached blades are located at 90 mm from the center of the axis as shown in Fig. 5. The dimensions are scaled down by a factor of 10 from a typical jet engine dimensions due to the limitation of the simulation while ensuring its accuracy. The scaled frequency used in the simulations is 5 GHz. The source of EM fields (excitation) in the design is provided by the simulation environment. An incident Hertzian dipole excitation (a) represents a simple transmitting antenna provided by HFSS full wave simulation environment simulates the field of an elementary short dipole antenna. The proposed model cut down the complexity of the jet engine problem. However, this simplified model gives a profound judgment into the EM propagation characteristics inside complex jet engine cavity^13,14^.

**Figure 6:**
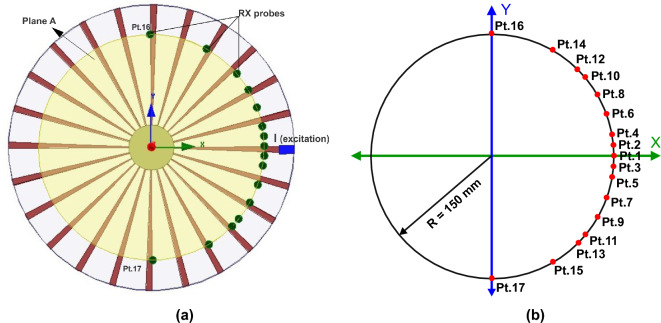
(**a**) Front view of jet engine simulation model showing circular Plane ${\textbf{A}}$, Rx probes over a circle, and excitation (I) location. (**b**) Schematic representation of location of Rx probes over a circle on circular Plane ${\textbf{A}}$.

In order to ensure the randomness inside the jet engine environment, the blades (stirrers) are simulated for 120 distinct position with an angular rotation step of 0.125$^\circ$. The characteristics of the field values are analyzed at a receiving circular plane (Plane ${\textbf{A}}$), which is positioned 6.6 cm away from the central axis along the $-Z$ direction as shown in Fig. 5b^13,14^. In order to have suitable statistical modeling of the attributes of the EM field, there should be at least $\lambda /4$ distances between the receiver and the conducting wall of the complex metallic environment. To mitigate this assumption, receiver (Rx) probes are positioned at $\theta =\pm \,90\,^\circ , \pm \,60\,^\circ , \pm \,45\,^\circ , \pm \,40\,^\circ , \pm \,30\,^\circ , \pm \,20\,^\circ , \pm \, 10\,^\circ , \pm \, 5\,^\circ$, and $0\,^\circ$ over a circle which has a 150 mm ($\lambda /4$) radius on plane ${\textbf{A}}$ as shown in Fig. 6^14^.

The magnitude of the electric field values ($E_{DZ}$) is extracted for 120 different blade rotational positions at those fixed 17 receiving points. Then, we average the field values to compare with the statistical concepts. The analysis proved that the squared magnitude of the rectangular electric field component; ${|E_{DZ}|}^2$ follows an exponential distribution, when the antenna is at $\lambda$ distance from the center of the axis^13,28^. The dependence on Z will be omitted in the following sessions, since all our results are extracted along Z axis. Figure 6a shows the location of these Rx points on the circular Plane ${\textbf{A}}$ along with the location of the transmitter (represented as Tx) inside the jet engine geometry. From Fig. 6a,b, it is clear that there is geometrical symmetry exist between the Rx probe locations with respect to the Tx. The distribution of the field inside the environment is due to the contribution of all the resonant modes that are available inside the jet engine environment. However, each and every mode will contribute in different style at different parts of the engine and hence, the strength and orientation of the electric field at different receiving points will be different even if they are located symmetrically around the axis. However, if we consider the statistical properties (mean and standard deviation) of the average electric field values at those points, we can see that the concurrent Rx locations at clockwise and counter clock wise directions are statistically equivalent due to the geometrical symmetry inside the jet engine environment.

As we explained, the dynamic jet engine analysis is extremely tedious and time consuming. Thus, to reduce the complexity of the analysis, we considered some specific Rx probe locations. These specific positions are chosen in order to closely resemble the positions of the future Rx antenna locations. Thus, for the following system analysis we use Rx probes that are located between $\theta$ = 0$^\circ$–45$^\circ$ only as representative to the whole semicircle, due to the symmetry inside the environment. In the near future, we will choose the best Rx location from these Rx probe locations depending upon the field strength.

#### Verification of DS mathematical model using DSS

To validate (5), a simplified jet engine simulation model as explained in the previous session is analyzed for varying dimensions. The $\textbf{segment 1}$ is simulated for different values of $R_{r}$, varying from 190 to 170 mm. For each value of $R_{r}$, the simplified jet engine model is simulated for 120 different blade positions and the magnitude of the electric field values is extracted at particular receiving positions located from $\theta$ = 0$^\circ$ to 90$^\circ$. At each receiving point, the electric field values are analyzed statistically to evaluate the field characteristics. The analysis proved that the squared magnitude of the rectangular electric field component; ${|E_{D}|}^2$ follows an exponential distribution, for all jet engine dimensions^13,28^. Then, the variation of $\sigma$ of electric field with varying dimension is also analyzed statistically.

**Figure 7:**
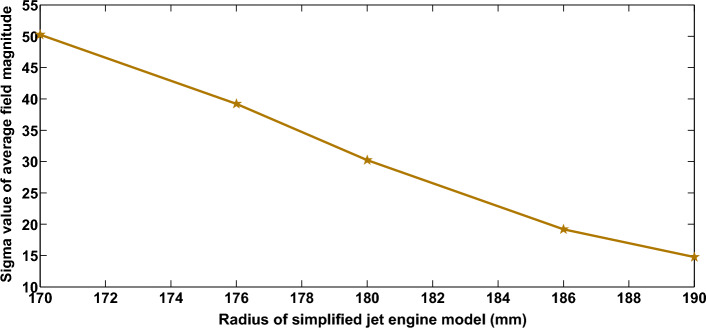
Relation between standard deviation and radius of the jet engine at a fixed receiver location.

Figure 7 proves the relation between the radius of jet engine environment and the standard deviation at a fixed Rx location. It is clear from Fig. 7 that as the radius of a jet engine increases the standard deviation decreases and is applicable to all other Rx probe locations, since jet engine environment is isotropic^28,39^.

This results validate our dimension scaling method analysis and proves that the $\sigma$ of the field magnitude is inversely related to the radius of the jet engine segment as in (5). This means that it is possible to analyze the entire jet engine environment by using DSA method, where a combination of dynamic simulation and the dimension scaling approach are used for the analysis of EM propagation through the complex jet engine environment.

However, as explained before the efficiency of the dynamic system analysis requires an increased simulation positions and at the same the concept of confidence interval study of statistical parameter is also required. Hence, to reduce the system complexity the SSA is introduced.

### *Approach-2*: static system approach

As explained in the previous section, SSA is an alternative approach to the DSA method, which introduces a novel SE method to solve the EM propagation through the entire jet engine geometry. The SSA characterize the **segment 1** with fixed blade and random excitation. In order to randomize the environment a Gaussian distributed excitation is used to illuminate the simplified jet engine model as shown in Fig. 5.

Initially, a 120 samples of Gaussian distributed random excitation; $\Delta a$ for the static system is generated by using (13) and (17). Later, the small signal equation is used to generate the values of $E_{s}(a+\Delta a)$ as in (10) for all the 120 random excitation. The magnitude of the output electric field values, $E_{s}(a+\Delta a)$, are evaluated at some particular Rx points and are analyzed statistically. The $\mu _{S}$, and $\sigma _{S}$ of average electric field values are generated using numerical approach.

**Figure 8:**
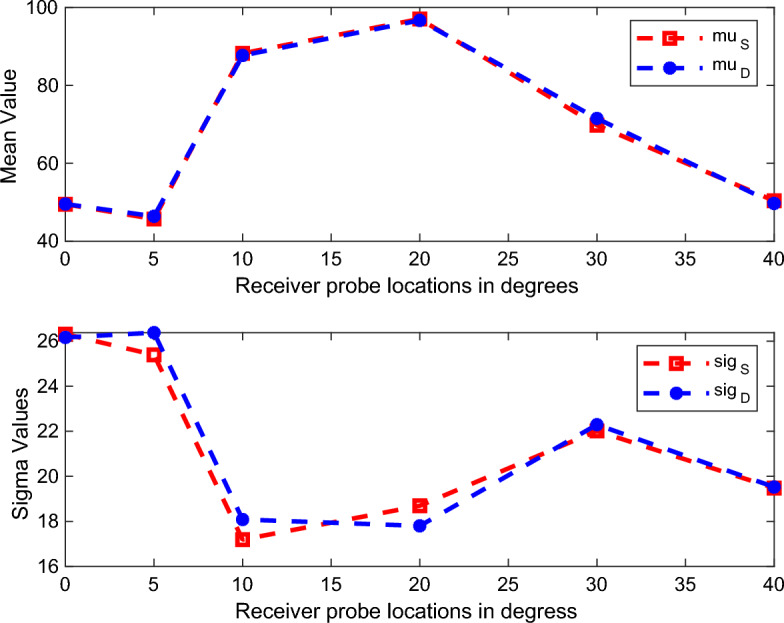
Comparison of statistical parameters ($\mu$ and $\sigma$) of the dynamic system (D) and static system (S) computed by HFSS and numerical method; respectively.

Figure 8 represents the comparison of the statistical parameters of dynamic and static system model; ie; $\mu _{D}$ and $\mu _{S}$, $\sigma _{D}$ and $\sigma _{S}$. It is important to mention that the dynamic system parameters are due to the dynamic blade rotation inside the simulation environment where the static system parameters are evaluated using numerical approach. It is clear from figure that the two systems almost have same mean and variances and hence proved that the static jet engine environment is statistically equivalent to the dynamic system. Furthermore, this analysis proved that SE method can replace the complex simulation analysis method to analyze the EM characteristics inside the jet engine environment. It is important to mention that the SE method which analyze the **segment 1** numerically, helps to eliminate the complex dynamic simulations.

#### Validation of the static system using simulation approach

**Table 1:** Comparison of statistical parameters generated using numerical and simulation approach of the static system model.

RX location	$\mu _{s}$ (numerical)	$\mu _{S}$ (FEM simulation)	$\sigma _{s}$ (numerical)	$\sigma _{S}$ (FEM simulation)	TX location
Pt$_1$	49.5400	48.0100	26.3109	26.5577	190-45
Pt$_2$	45.6944	47.8133	25.3916	27.4004	190-45
Pt$_1$	72.9893888	73.5258	25.25011652	24.5119	190-75
Pt$_2$	56.08390719	55.9224	27.2703864	26.6964	190-75
Pt$_4$	61.21927607	61.9095	28.90034691	31.6079	190-75

As mentioned in “Introduction”, the available experimental data are collected for systems not matching our considered system model. Consequently, these data are unsuitable for validating our proposed SE model. As a result, the attributes of the output field values of the static system, assessed through a numerical methodology, are validated with FEM simulation. To validate our numerical results derived from the proposed static system, a statistical excitation system model is simulated with a stationary blade position and a random current source as an excitation ($a+\Delta a$). However, the random excitation at each Rx location has different mean and variances. Thus, to simulate the whole Rx location we need to generate random excitation using corresponding mean and variances. $\mu _{\Delta }$ and $\sigma _{\Delta }$ for each Rx points are calculated using (13) and (17). 120 random samples of the Gaussian distributed $\Delta a$ are generated using the calculated mean and variance corresponding to each Rx probe location. Then, the total Gaussian distributed random excitation current for the static system is generated as in (6). Later, the static system is simulated for 120 random excitations which provide the necessary randomness inside the environment. The electric field, $E_{s}(a+\Delta a)$, is extracted for that particular Rx location and average field values over 120 random excitations are analyzed statistically. Later, the statistical parameters such as $\mu _{S}$ and $\sigma _{S}$ of the static system from FEM simulation environment also evaluated to verify our proposed numerical approach method.

Table 1 represents verification of the numerical approach used for the evaluation of statistical parameter of the static system using FEM simulation method. The static system parameters are extracted by using HFSS simulation method and compared with the parameters evaluated using the numerical approach (from (10)). It is clear from the table that the two systems almost have the same mean and variances. Hence, the numerical results are validated with the help of simulation analysis. Since the FEM simulation is extremely complex and takes prohibitively long time to produce results, we opted for validation at few points only; specially that we have a mathematical derivation of the statistical equivalency between the static and dynamic systems. The verification points were carefully selected to incorporate a mix of change in system parameters, i.e., varieties of TX and RX locations.

#### Sensitivity of analysis to main system parameters

**Figure 9:**
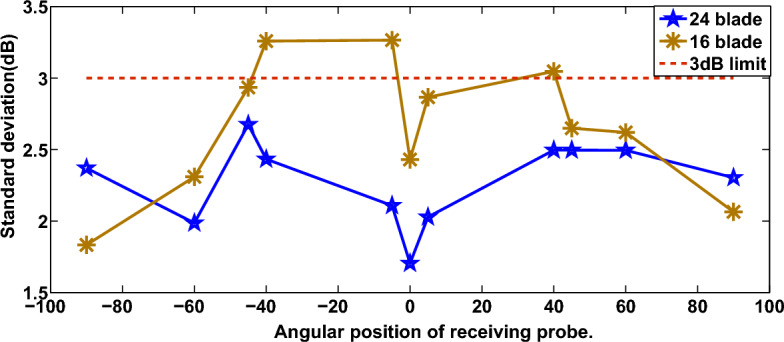
Field uniformity with different number of rotating blades^28^.

Referring to (5), it can be noticed that the geometry of the jet engine cavity’s radius ($R_{r}$) affects the variance of electric field. Moreover, we show in Fig. 9 the effect of number of blades on the uniformity of electric field distribution, where the uniformity refers to the variation of the standard deviation around the mean^28^.

## Discussions

**Figure 10:**
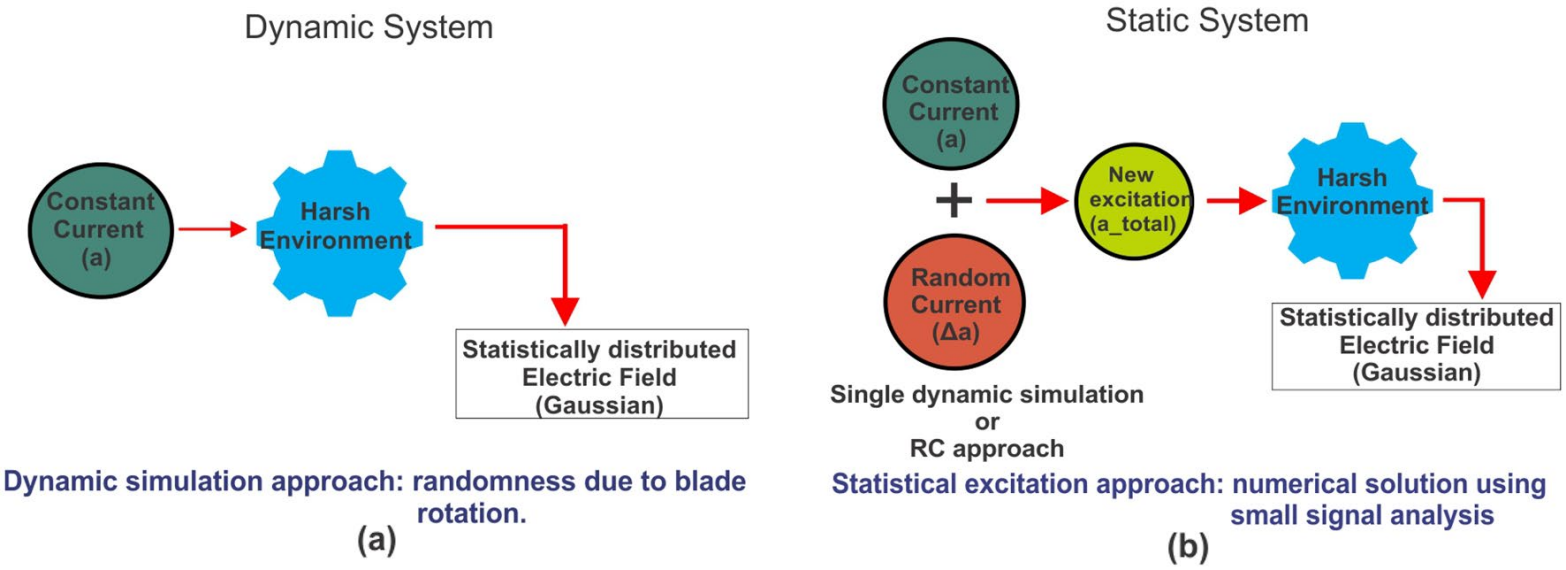
Block diagram representation of comparison between proposed system models. (**a**) Dynamic system. (**b**) Static system.

In this journal, we proposed statistical electromagnetics as an exceptional tool that can capture the time-dynamic effects of the jet engine environment while providing feasible and accurate results compared to the classical deterministic approach. Moreover, this method is sufficiently accurate yet simple enough to describe the fields inside the complex and dynamic cavity environments using the statistical methods. To enhance the efficiency and precision of EM propagation analysis within a complex jet engine system, we introduce two distinct methodologies. The dynamic system approach analyzes the behavior of EM propagation through dynamic jet engine system, where the randomness is provided by the rotation of the blades. The dynamic approach still requires a substantial number of simulation procedures, thereby augmenting the complexity of the analysis. Additionally, we introduce the concept of the static system as a method to transform the source of randomness within a dynamic jet engine, stemming from blade rotation, into randomness resulting from the random variation of excitation.This new proposed approach aids in simplifying the analysis while maintaining accuracy. The proposed approaches can be summarized as shown in Fig. 10. Figure 10a shows the block diagram of the proposed dynamic system model where the randomness is due to repeated blade rotation. Figure10b shows the block diagram of the proposed static system model where the randomness due to repeated blade rotation is translated as random excitation.

As we discussed before, the analysis of the EM propagation through jet engine has evolved from the classical approaches to the statistical electromagnetic approach. The complete analysis of a complex system like jet engine environment using classical deterministic approach is exceptionally problematic because of the dependency of the analysis to the details of the cavity’s dimensions, rotating metallic parts, material property etc. A comparison of existing classical approaches with our proposed statistical approach is provided in the Table 2. As shown in Table 2, the main weakness of the classical approaches is their inability to capture the time dynamics resulting from the rotation of the metallic parts (the blades). This was the main motivation for our work.

**Table 2:** Comparison of proposed approaches with classical approaches.

Proposed		Classical	
DynamicSystem	StaticSystem	Simulation	Analytical
A statistical analysis based on repeated simulations with blades step rotation with very high computational complexity	A statistical analysis based on random excitation and numerical solution with low computational complexity	Deterministic approach that cannot capture the rotational property of the configuration with medium computational complexity	Deterministic approach that cannot capture rotational property of the configuration with very less computational complexity

Applying our proposed EM models to diverse and complex jet engines introduces several challenges and limitations that necessitate careful consideration. The dynamic system offers a detailed analysis of jet engine environment by incorporating a multitude of simulations, it comes at the cost of prolonged computation times due to the intricate geometric setup involving blades, rotors, and stators. The dynamic nature of the jet engine environment and the extremely large size of the geometric configuration of the jet engine influences the simulation model. Large sizes can lead to computational challenges and may require simplifications or scaling to make the simulation feasible. Thus, we developed a scaled jet engine model to reduce the computational complexity of the simulation environment. The primary potential constraint in our proposed static system approach lies in the elimination of higher order terms from the Taylor expansion, as elucidated in the mathematical modeling of the static system. The exclusion of higher order terms from the Taylor equation, a key step in constructing the small signal model, may introduce inaccuracies in the static system analysis, particularly when applied to high-power systems.

## Conclusions

In this paper, the propagation characteristics of EM field inside the jet engine environment are analyzed using statistical electromagnetic approach. It is important to mention that this method is sufficiently accurate, yet simple enough to describe the fields inside the complex and dynamic cavity environments compared to the classical deterministic perspective. Two different models are proposed; simulation based dynamic system and mathematical based static system, to model the EM characteristics inside the jet engine environment. A simple segmentation approach is proposed to reduce the complexity of the jet engine geometry and the procedure of the analysis for both dynamic and static cases are described in detail. The proposed dynamic system consists The dynamic system is analyzed using HFSS by varying the position of the blades 120 times to simulate rotation effect of blades which provide the necessary randomness inside the medium. The magnitude of the electric field is extracted and analyzed to obtain the statistical parameters of the dynamic system, which are used to set the parameters of the static system in order to establish statistical equivalence between the two systems. The static system model is represented by a fixed blade position while the excitation is randomized. The excitation randomization is obtained using a Gaussian distributed 120 random excitations. Small signal equation is used to generate the values of static electric field for all the 120 random excitation. Result of the numerical analysis of the static system model agrees well with the result of FEM simulation results. Numerical results demonstrate the reliability of the proposed SE technique. The statistical equivalence between the two systems has been verified. The effect of jet engine dimensions on the field characteristics is analyzed statistically and proved that $\sigma$ of the electric field value varies linearly in varying dimensions.Since the static system is significantly computationally simpler, the proposed static approach will help in analyzing complex dynamic systems such as jet engines without the need for repeated complex simulations stemming from blade rotations.

## Challenges and future work

As explained before, the ultimate objective of this particular research is to study the nature of electromagnetic propagation through complex metallic environments so that a communication link can be established inside the jet engine environment. Thus, we proposed statistical electromagnetic as a potential alternative to existing numerical approaches to characterize the EM propagation through jet engine environment. The proposed static system approach also eliminates the need of complex multiple simulations to solve the EM propagation through jet engine environment. However, there are different challenges are existing in implementing this proposed approaches in real world scenario such as (1) need more comprehensive study in terms of wireless communication channel modeling. (2) Experimental work is necessary to further verify and implement this analysis. While the proposed method has shown promising outcomes, it is important to validate the reliability of proposed approaches through comparison against experimental data. To the best of our knowledge, the availability of experimental data for a full-size engine model is scarce due to the fact that acquiring real jet engine is challenging due to cost and industrial confidentiality concerns. Moreover, the very few available literature that use experimental work are dealing with different objectives and system configurations. Hence, the comparison of our model with existing work in literature was not possible. As a potential future alternative, experimental data can be attained through two alternative approaches. A small size commercial jet engine model can be procured to perform validation through comparing simulation with experimental measurements. A second alternative would be building a mock scaled down jet engine framework to emulate the key propagation environment (rotor frame, stator frame and blades). This model can be used to obtain experiment measurements to be compared with analysis and simulation results. The future analysis may also involve an in-depth analysis of a multistage rotor and stator jet engine model, employing EM simulation and experimental modeling to explore the EM wave propagation within a highly realistic jet engine environment.

## Data Availability

Correspondence and requests for materials should be addressed to A.K. or T.K.
